# Machine learning slice-wise whole-lung CT emphysema score correlates with airway obstruction

**DOI:** 10.1007/s00330-023-09985-3

**Published:** 2023-08-08

**Authors:** Mats Lidén, Antoine Spahr, Ola Hjelmgren, Simone Bendazzoli, Josefin Sundh, Magnus Sköld, Göran Bergström, Chunliang Wang, Per Thunberg

**Affiliations:** 1 Department of Radiology and Medical Physics, Faculty of Medicine and Health, Örebro University, 701 82 Örebro, Sweden; 2 Department of Biomedical Engineering and Health Systems, KTH Royal Institute of Technology School of Technology and Health, Stockholm, Sweden; 3 Department of Molecular and Clinical Medicine, Institute of Medicine, Sahlgrenska Academy, University of Gothenburg, Gothenburg, Sweden; 4 Department of Clinical Physiology, Sahlgrenska University Hospital, Region Västra Götaland, Gothenburg, Sweden; 5 Department of Clinical Science, Intervention and Technology – CLINTEC, Karolinska Institutet, Stockholm, Sweden; 6 Department of Respiratory Medicine, Faculty of Medicine and Health, Örebro University, Örebro, Sweden; 7 Department of Medicine Solna, Karolinska Institutet, Stockholm, Sweden; 8 Department of Respiratory Medicine and Allergy, Karolinska University Hospital, Stockholm, Sweden

**Keywords:** Tomography, X-ray computed, Pulmonary emphysema, Pulmonary disease, chronic obstructive, Lung, Deep learning

## Abstract

**Objectives:**

Quantitative CT imaging is an important emphysema biomarker, especially in smoking cohorts, but does not always correlate to radiologists’ visual CT assessments. The objectives were to develop and validate a neural network-based slice-wise whole-lung emphysema score (SWES) for chest CT, to validate SWES on unseen CT data, and to compare SWES with a conventional quantitative CT method.

**Materials and methods:**

Separate cohorts were used for algorithm development and validation. For validation, thin-slice CT stacks from 474 participants in the prospective cross-sectional Swedish CArdioPulmonary bioImage Study (SCAPIS) were included, 395 randomly selected and 79 from an emphysema cohort. Spirometry (FEV1/FVC) and radiologists’ visual emphysema scores (sum-visual) obtained at inclusion in SCAPIS were used as reference tests. SWES was compared with a commercially available quantitative emphysema scoring method (LAV950) using Pearson’s correlation coefficients and receiver operating characteristics (ROC) analysis.

**Results:**

SWES correlated more strongly with the visual scores than LAV950 (*r* = 0.78 vs. *r* = 0.41, *p* < 0.001). The area under the ROC curve for the prediction of airway obstruction was larger for SWES than for LAV950 (0.76 vs. 0.61, *p* = 0.007). SWES correlated more strongly with FEV1/FVC than either LAV950 or sum-visual in the full cohort (*r* =  − 0.69 vs. *r* =  − 0.49/*r* =  − 0.64, *p* < 0.001/*p* = 0.007), in the emphysema cohort (*r* =  − 0.77 vs. *r* =  − 0.69/*r* =  − 0.65, *p* = 0.03/*p* = 0.002), and in the random sample (*r* =  − 0.39 vs. *r* =  − 0.26/*r* =  − 0.25, *p* = 0.001/*p* = 0.007).

**Conclusion:**

The slice-wise whole-lung emphysema score (SWES) correlates better than LAV950 with radiologists’ visual emphysema scores and correlates better with airway obstruction than do LAV950 and radiologists’ visual scores.

**Clinical relevance statement:**

The slice-wise whole-lung emphysema score provides quantitative emphysema information for CT imaging that avoids the disadvantages of threshold-based scores and is correlated more strongly with reference tests than LAV950 and reader visual scores.

**Key Points:**

• *A slice-wise whole-lung emphysema score (SWES) was developed to quantify emphysema in chest CT images.*

• *SWES identified visual emphysema and spirometric airflow limitation significantly better than threshold-based score (LAV950).*

• *SWES improved emphysema quantification in CT images, which is especially useful in large-scale research.*

**Supplementary Information:**

The online version contains supplementary material available at 10.1007/s00330-023-09985-3.

## Introduction

Chronic obstructive pulmonary disease (COPD) is the third leading cause of death worldwide [1]. The main symptoms are shortness of breath, exertional dyspnea, and cough. COPD is mainly caused by cigarette smoking that induces a variable combination of bronchiolitis and emphysema resulting in chronic airflow limitation. Chronic bronchitis, i.e., cough with phlegm, may also be present.

Emphysema is characterized by destruction of alveolar walls with impaired gas exchange and hyperinflation, visualizable on chest CT images as low-density regions [2]. COPD is irreversible and should be diagnosed as early as possible to prevent progress. The diagnosis is confirmed by spirometry (pulmonary function testing, PFT) revealing chronic airway obstruction not normalizable with bronchodilators or other therapy [3].

Computed tomography is the modality of choice for emphysema visualization. The extent of emphysema can be estimated by a radiologist, but quantitative image information is desirable, for example, as outcome in clinical trials [4, 5]. Quantitative and visual CT image scores add information to PFT and help predict morbidity and mortality in COPD, independent of PFT results [6–10].

The Swedish CArdioPulmonary bioImage Study (SCAPIS) is a multi-center, cross-sectional study including chest CT scans and spirometry for over 30,000 individuals. The aim of SCAPIS is to predict and prevent cardiovascular disease and COPD [11]. Inter-observer variation in CT evaluation is a well-known challenge in clinical trials [12]. For multi-center trials, such as SCAPIS, an unbiased analytical tool for core-lab can reduce inter-observer variation.

The fraction of low-attenuation pixels in the lungs, i.e., low-attenuation volume below − 950 HU (LAV950), is a frequently used quantitative CT emphysema metric in COPD and smoking cohorts [6, 7, 13–16]. However, LAV950 correlates weakly with visual emphysema scores in cross-sectional cohorts [17]. Furthermore, even in prediction models that include LAV950, the visual emphysema score remains a significant predictor, suggesting that LAV950 captures only part of the CT image information [8].

In this study, we aim to create a refined reader-independent quantitative emphysema metric for CT images that measures what radiologists identify as emphysema. Using machine learning and detailed radiologist emphysema annotations, we introduce a slice-wise whole-lung CT emphysema score (SWES) to combine the predictive value of visual scores with the objective assessment of quantitative CT.

The purposes of the current study were (a) to develop a machine learning SWES method for lung CT; (b) to externally validate the method against radiologists’ emphysema scoring on unseen CT data with the test hypothesis of better correlation with radiologists’ scores for SWES compared to LAV950; and (c) to compare the correlation with PFT between the SWES method, a commercial LAV950 application, and visual emphysema scoring.

## Materials and methods

**Fig. 1:**
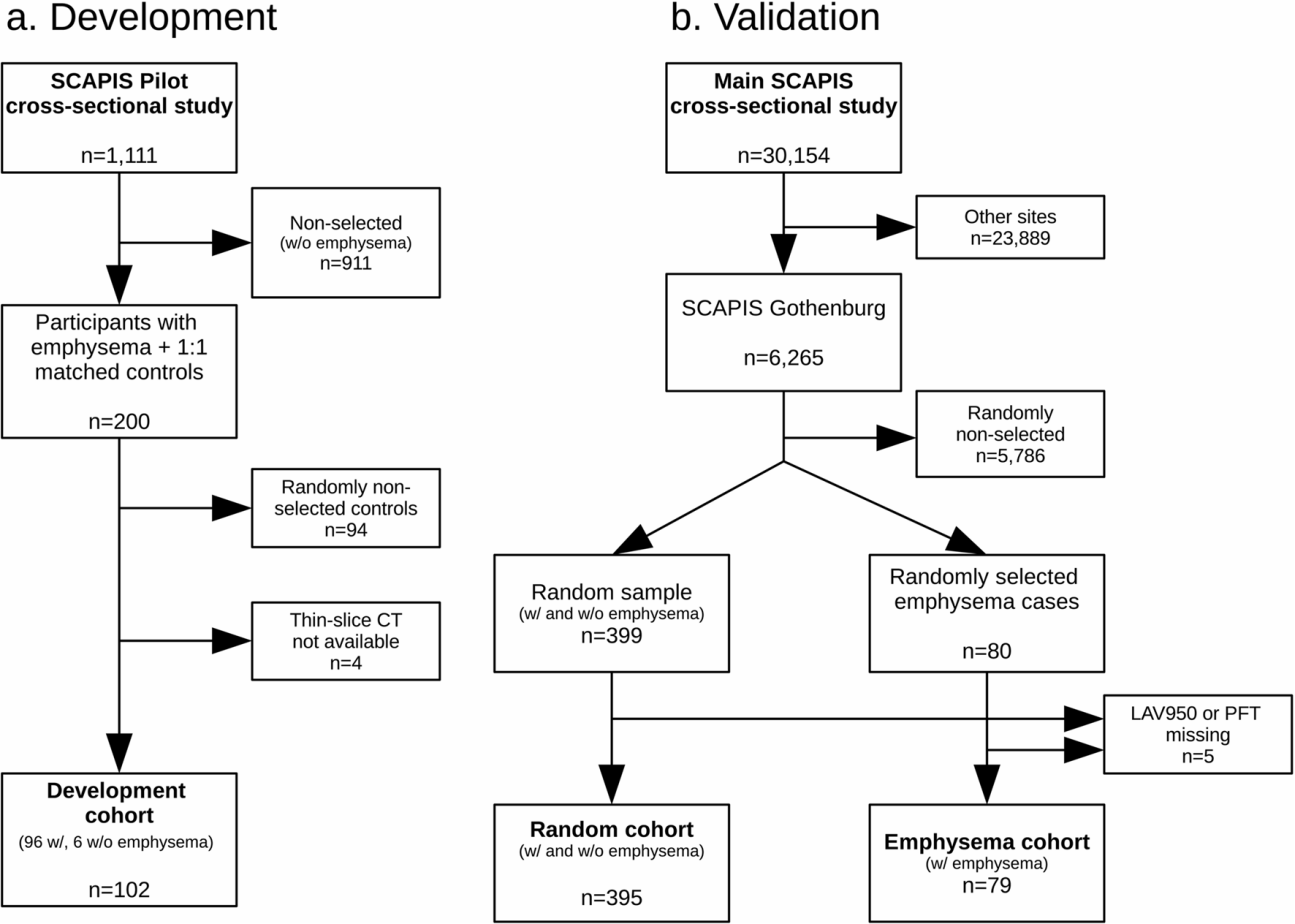
Inclusion flowchart. **a** Development cohort. Only six of the matched controls were selected to avoid worsening the already skewed distribution of regional emphysema scores. **b** The total validation cohort (*n* = 474) consisted of a random (*n* = 395) and emphysema (*n* = 79) cohort. LAV950, low-attenuation volume − 950 HU; PFT, pulmonary function testing

The Swedish Ethical Review Authority approved the study protocol. Written informed consent was obtained at inclusion. SCAPIS prospectively included 30,154 participants at six Swedish university hospitals including Gothenburg from 2013 to 2018. A pilot study, SCAPIS Pilot, recruited 1111 participants in 2012 [17]. In this study, the development data originated from SCAPIS Pilot, while the external validation was performed on data from the main SCAPIS Gothenburg cohort. The inclusion process is described in Fig. 1.

In the present work, clinical terminology is used, defining external validation as the application of the machine learning method on the unseen CT data. The machine learning term test set is used only for the limited test set used during algorithm development.

### Image data

The image data were thin-slice unenhanced CT image stacks capturing the lungs at full inspiration, approximately 500 slices per examination. All images were acquired on a Siemens Somatom Definition Flash CT system. Acquisition parameters in development and validation cohorts were identical: 120 kVp, CARE dose quality reference mAs 25, pitch 0.9, rotation time 0.5 s, reference patient CTDI 1.7 mGy.

In the development cohort (Fig. 1a), the 102 CT stacks were reconstructed as 0.6-mm/0.6-mm (slice thickness/increment) slices. The reconstruction filters were a medium smooth soft tissue filtered back projection algorithm, B31f (*n* = 42), or medium smooth soft tissue iterative reconstructions (SAFIRE) strength 2/5 (I31f2, *n* = 12) or strength 3/5 (I31f3, *n* = 48).

In the validation cohort (Fig. 1b), all images were reconstructed as 0.75-mm/0.6-mm (slice thickness/increment) slices using a smooth soft tissue filtered back projection algorithm, B20f.

### Algorithm development

#### Detailed slice-wise annotations

The machine learning emphysema prediction model was developed using only thin-slice data from the SCAPIS Pilot cohort.

Detailed slice-wise emphysema annotations were acquired in a three-step process including multiple readers in the first (26 readers) and third steps (16 readers) (see supplemental Fig S1–S3). In the first step, each centimeter of each lung was classified according to a 4-degree emphysema scale as previously described [18]. The second step consisted of median *z*-direction filtering of the 4-degree annotations and the third step was a refinement algorithm, increasing the granularity of the emphysema labels from a 4-degree scale to 10 degrees (see Appendix A for details).

#### Machine learning slice-wise emphysema scoring (SWES) method

The 10-degree annotations in the development cohort were used in developing a machine learning method based on a convolutional neural network (CNN) and a linear regressor. The method processed a chest CT scan, with separate outputs from each slice and lung.

**Fig. 2:**
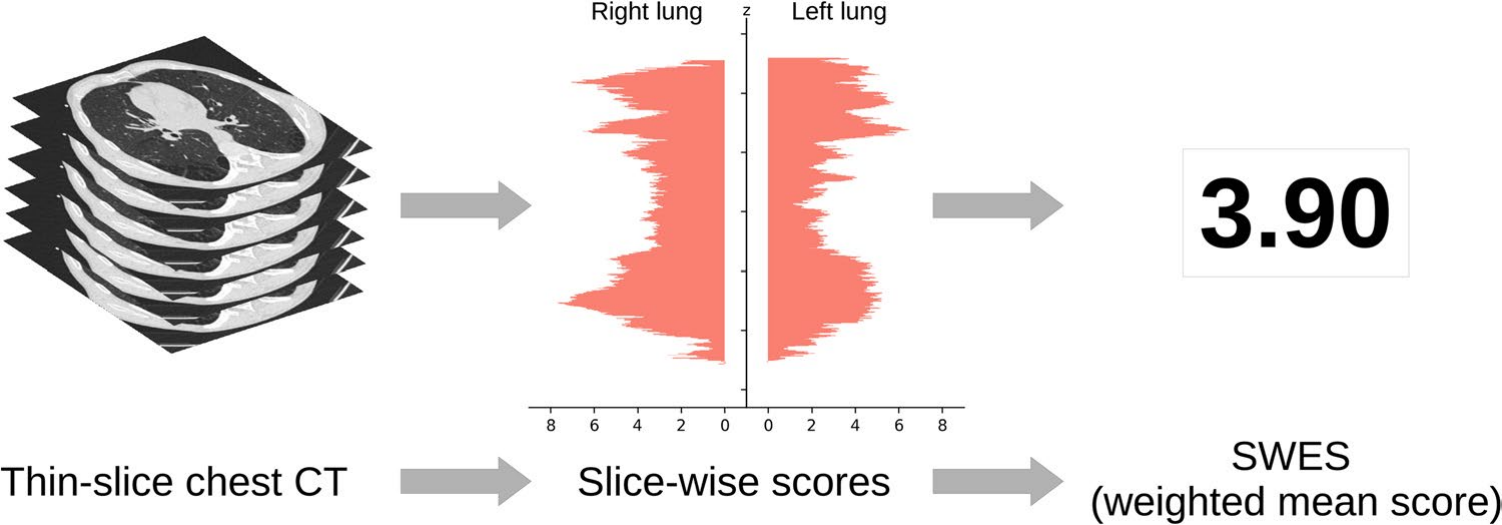
Slice-wise whole-lung emphysema score (SWES)

For each patient, the SWES was constructed as the average emphysema score for each lung and slice, weighted by the segmented lung area (see Fig. 2).

The intra-scan repeatability of SWES was analyzed on a subset of 63 CT stacks that were randomly rotated up to ± 7.5° in the *x*–*y*-, *x*–*z*-, and *y*–*z*-plane simultaneously. The Bland–Altman limit of agreement between SWES computed in the original and rotated image volume was computed.

#### Neural network and CT scan pre-processing

The neural network was trained on axial CT images with pre-processing intended to direct the training on the lung-texture features of emphysema and to reduce the imbalanced data containing few examples of advanced emphysema in the training cohort (Supplemental Figure S4). For prediction, only segmentation, contrast windowing, cropping, and resizing were used. Pre-processing details are given in Appendix B.

A ResNet-18 architecture was adopted with the final linear as a single neuron predicting the emphysema score for the slice [19]. From the 102 CT stacks in the development cohort, 82 were used for training/validation and 20 were held-out for testing. The binned accuracy was computed on the test data, where the outcome measure was fraction of predictions within ± 1.5 from slice emphysema annotation.

The regression network was optimized on the training set with oversampling to partially compensate the label imbalance (see supplemental Table S1). Optimization was performed using back-propagation and the Adam algorithm with default parameters. The loss function was the sum of the mean-square-error and the mean-absolute-error weighted by the inverse of the label proportion in the oversampled train dataset to balance rare labels.

The model was evaluated in each epoch and the final model was selected as the one displaying the lowest validation loss aiming to select a model with best fit on unseen data. The model was trained five times with different random seeds to assess the stability. Each time, the ResNet-18 was trained for 75 epochs with a batch size of 32 and a learning rate of 0.001 exponentially decayed with a power of 0.95 at each epoch. The network’s parameters were regularized with an L2 penalty with a weight of 10^−6^.

The source code for the slice-wise predictions is available as supplementary material.

### Validation reference metrics

The main Gothenburg SCAPIS cohort was used for validation, with electronic case report forms (eCRF) visual emphysema scoring and PFT for comparison. None of the validation data was used in algorithm development or training. The validation data consisted of two cohorts made available by SCAPIS: 395 randomly selected cases, and 79 selected cases of emphysema according to eCRF visual scores (see Fig. 1b). There was no overlap between the cohorts.

#### Pulmonary function testing

Each SCAPIS participant was tested using dynamic spirometry 15 min after inhaling 400 µg of salbutamol [11]. A post-bronchodilator-forced expiratory volume in 1 s (FEV1) divided by forced vital capacity (FEV1/FVC) ratio of under 0.7 confirms chronic airway obstruction compatible with COPD [3], and FEV1/FVC is also the PFT parameter with the strongest correlation with LAV950 and visual emphysema scoring [14, 20]. It was therefore chosen as validation reference. Correlations with post-bronchodilator carbon monoxide diffusing coefficient in percent of predicted (DLcoPred%), in participants with available data, were also used [11],

#### Visual scoring

Three regions in each lung were reviewed at inclusion in SCAPIS: upper, middle, and lower using a Syngo.Via (Siemens Healthineers) thin-slice workstation. In each region, emphysema was graded on a 4-point scale: none, mild, moderate, or severe [17]. In this study, the eCRF emphysema score in each region was coded 0–3, and the sum of codes for all regions was used as a patient score of 0–18 (sum-visual score).

Significant visual emphysema was defined as a sum-visual score > 2, corresponding to more than two regions with mild emphysema or more than one region with moderate emphysema.

#### LAV950

LAV950 was assessed using AI-Rad Companion Chest CT (Siemens Healthineers). The LAV950 analysis is threshold-based; the algorithm determines the fraction of all voxels below − 950 Hounsfield units (HU) in the lungs. The analysis was performed using a fully automated workflow without any manual adjustments. The automated results were verified with the segmentations in the SWES algorithm (see appendix C).

### Statistics

Pearson’s correlation coefficients between SWES and sum-visual were used to assess whether SWES measures what radiologists identify as emphysema, with Meng’s test for dependent correlation coefficients to test the hypothesis of stronger correlation for SWES compared to LAV950 [21].

The SWES, LAV950, and sum-visual scores were correlated to FEV1/FVC and DLcoPred% in the random (*n* = 395), emphysema (*n* = 79), and total validation (*n* = 474) cohorts, separately. Pearson’s correlation coefficients were compared using Meng’s test.

Receiver operating characteristic statistics were used for SWES and LAV950, considering prediction of significant visual emphysema, and airway obstruction, as defined by the GOLD criteria for COPD (FEV1/FVC < 0.7). ROC curves were compared using DeLong’s test and 95% CI were obtained through bootstrapping.

Statistics were computed with Matlab R2020a (The Mathworks) and STATA 17.0 (StataCorp LLC).

## Results

### Baseline characteristics

Baseline characteristics of included participants are presented in Table 1. The development and validation cohorts were different regarding PFT as well as CT emphysema metrics (SWES, LAV950, and sum-visual). Also, the reconstruction parameters used were different in the development cohort compared to the validation cohorts.

**Table 1:** Baseline characteristics and imaging parameters

	Development cohort	Validation (random sample)	Validation (emphysema cohort)	*p*-value
*N*	102	395	79	
Patient characteristics				
Female (%)	47 (46%)	222 (56%)	38 (48%)	0.12^e^
Age (years)	58 ± 5	58 ± 4	60 ± 4	0.001
BMI (kg/m^2^)	26 ± 5	27 ± 4	26 ± 5	0.21
Height (cm)	170 ± 10	172 ± 9	172 ± 9	0.14
Weight (kg)	76 ± 16	79 ± 15	76 ± 17	0.07
LAV950 (%)	5 [2–9]	6 [3–9]	8 [4–13]	0.003
SWES (arb units)	0.8 [0.7–1.3]^a^	1.2 [1.0–1.5]	1.7 [1.2–2.7]	< 0.001
Sum-visual	2 [2–4]	0 [0–0]	4 [2–7]	< 0.001
FEV1/predicted (%)	97 ± 16^b^	108 ± 15	92 ± 21	< 0.001
FEV1/FVC	0.72 ± 0.09^b^	0.78 ± 0.06	0.67 ± 0.13	< 0.001
DLcoPred% (%)	n/a	93 ± 14^c^	77 ± 20^d^	< 0.001^f^
Image parameters				
CT scanner	Siemens Definition Flash	Siemens Definition Flash	Siemens Definition Flash	
Dose modulation	Care Dose 4D	Care Dose 4D	Care Dose 4D	
Reference mAs (ref-mAs)	25	25	25	
Tube potential (kVp)	120	120	120	
Slice thickness	0.6 mm	0.75 mm	0.75 mm	
Slice increment	0.6 mm	0.6 mm	0.6 mm	
Reconstruction algorithm	B31f (*n* = 42), I31f2 (*n* = 12), I31f3 (*n* = 48)	B20f	B20f	

Values are given as mean ± standard deviation or median [inter-quartile range]. *n/a*, not available

*B31f*, medium smooth soft tissue filtered back projection (FBP) algorithm; *B20f*, smooth soft tissue FBP algorithm; *I31f2*, *I31f3*, medium smooth soft tissue iterative reconstruction algorithms (SAFIRE, strength 2 and 3 out of 5, respectively). *p*-values are given for Kruskal–Wallis, except ^e^chi-squared test and ^f^Wilcoxon rank-sum test, as applicable

^a^Using SWES segmentations

^b^*n* = 101

^c^*n* = 328

^d^*n* = 61

#### Algorithm development

The slice-wise predictions on the 10-degree scale in the development cohort were stable on the unseen test set of 20 CT stacks not used in training. The model reached a slice-wise mean binned accuracy ± 1.5 of 83.7% over the replicates on the training-validation set, and 82.5% on the held-out test set.

Intra-scan repeatability comparing SWES on original and randomly rotated CT scans showed narrow limits of agreement, 0.06 ± 0.11.

#### Validation against visual emphysema

**Fig. 3:**
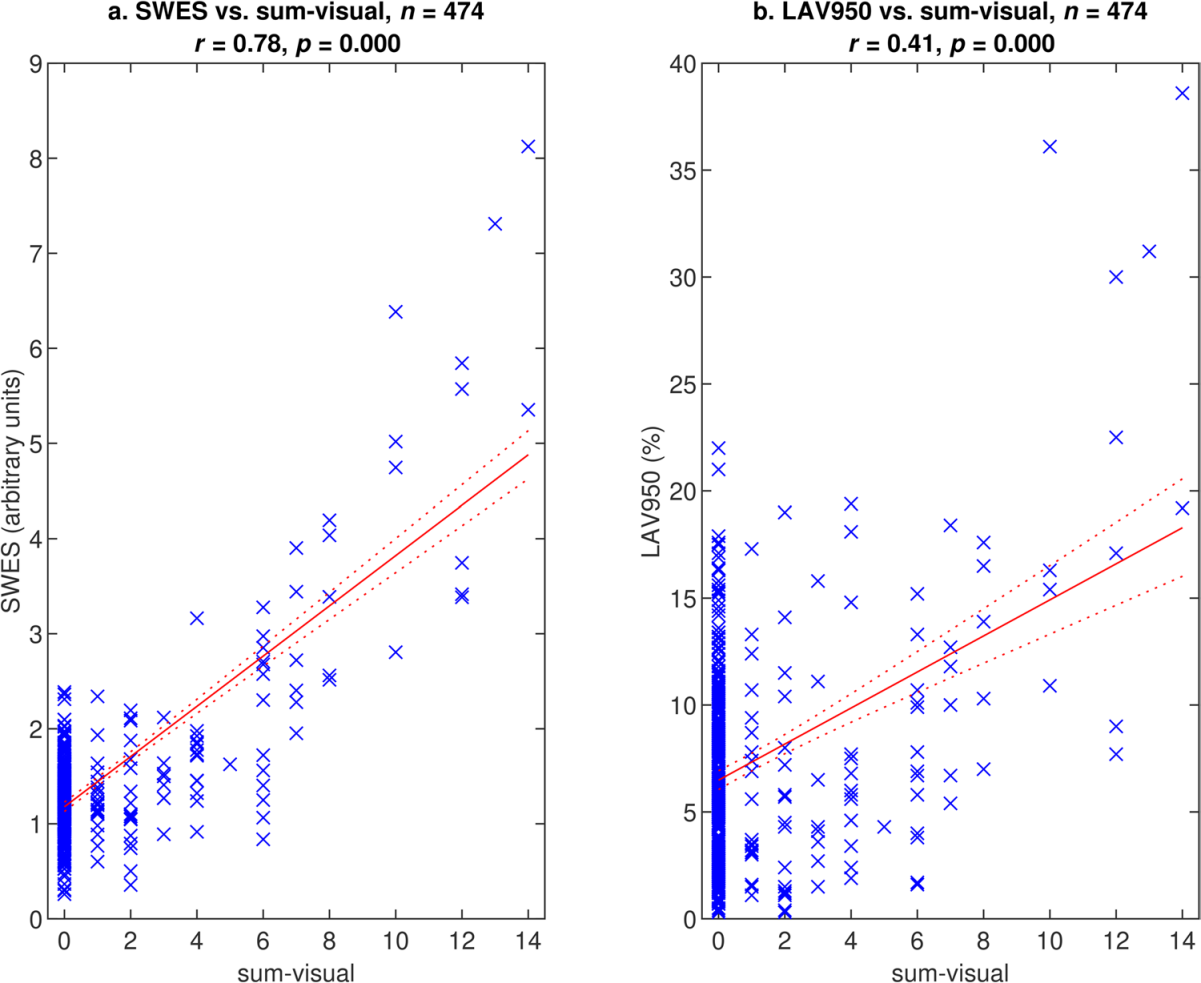
(**a**) Slice-wise whole-lung emphysema scores (SWES) and (**b**) low-attenuation volume below − 950 HU (LAV950) vs. sum of visual regional emphysema scores. Solid/dashed lines — regression line with 95% confidence bounds

**Fig. 4:**
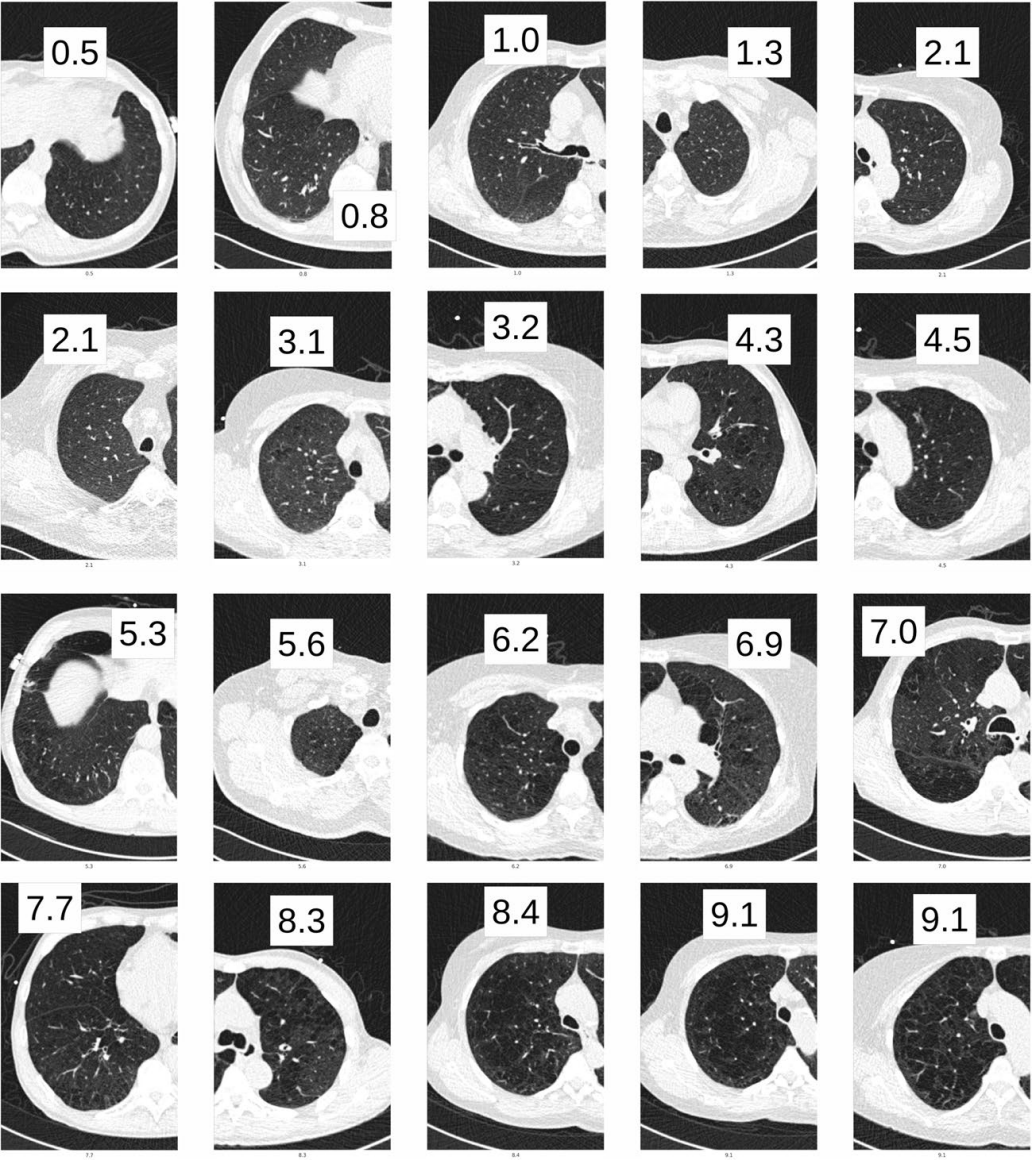
Examples of per-slice predictions in the validation dataset. The slices and sides are randomly selected from multiple participants to obtain uniformly distributed examples with scores between 0 and 10. Inserted numbers show per-slice emphysema score for shown lung. All images are shown in window level/width − 500/1200 HU

In the total validation cohort, SWES correlated strongly with the sum-visual regional score, while LAV950 correlated weakly (*r* = 0.78 vs. *r* = 0.41, *p* < 0.001 for difference) (see Fig. 3). The strong correlation indicates that SWES measures what radiologists identify as emphysema. Figure 4 shows the gradual increase in emphysema in randomly chosen slices with slice scores distributed between 0 and 10.

**Fig. 5:**
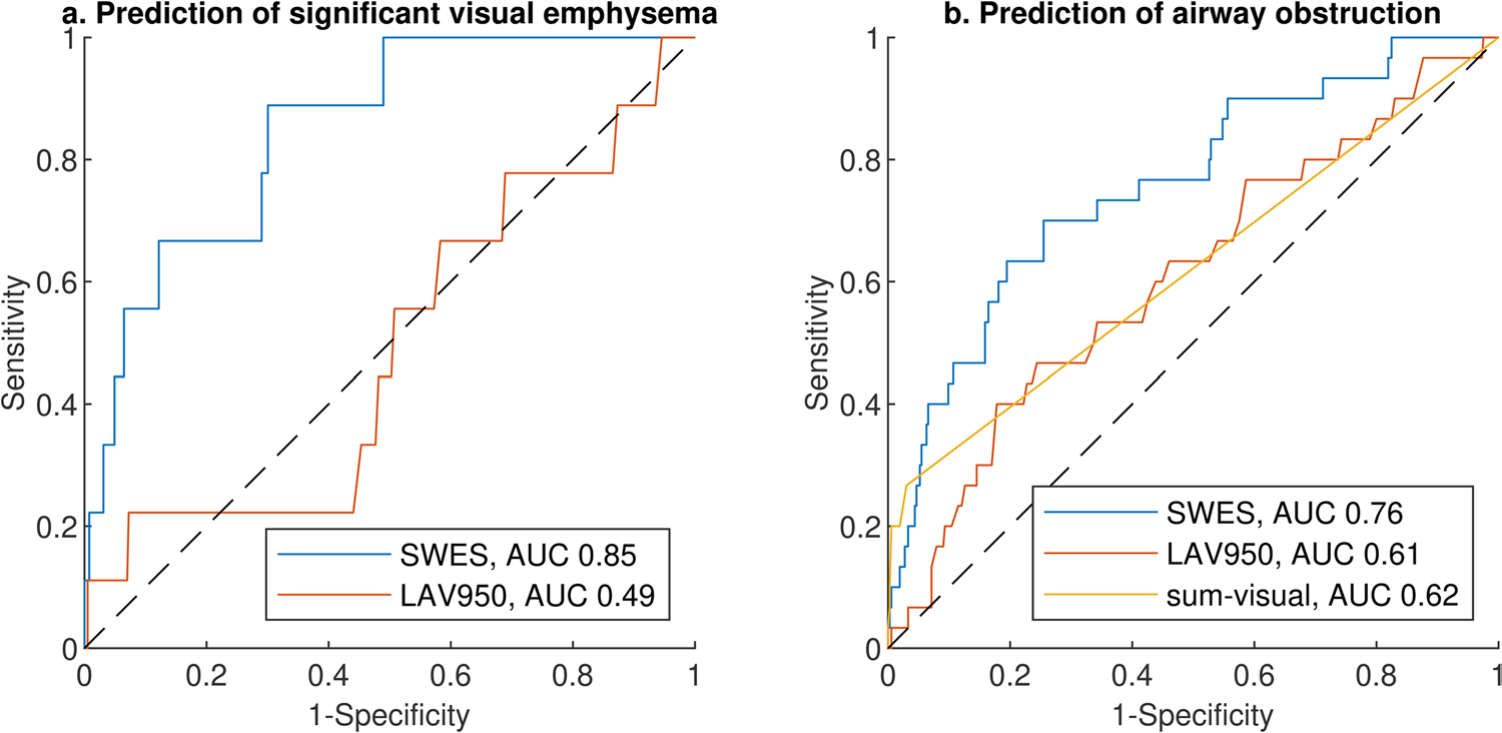
ROC curves. **a** Prediction of significant visual emphysema, defined as sum-visual > 2. The AUC for SWES was higher than that for LAV950 (*p* < 0.001). **b** Prediction of airway obstruction, defined as FEV1/FVC < 0.7. The AUC for SWES was higher than that for LAV950 (*p* = 0.007) and for sum-visual (*p* = 004). SWES, slice-wise whole-lung emphysema score; LAV950, low-attenuation volume below − 950 HU

With an area under the curve of 0.85 (95% confidence interval (CI) 0.74–0.96), SWES was an excellent predictor of significant emphysema (sum-visual > 2) in the random cohort, while LAV950 did not discriminate between cases with and without significant emphysema (AUC 0.49 (95% CI 0.29–0.70) (*p* < 0.001 for difference)) (see Fig. 5a).

#### Correlation with PFT compared to LAV950 and sum-visual

##### Airway obstruction

**Fig. 6:**
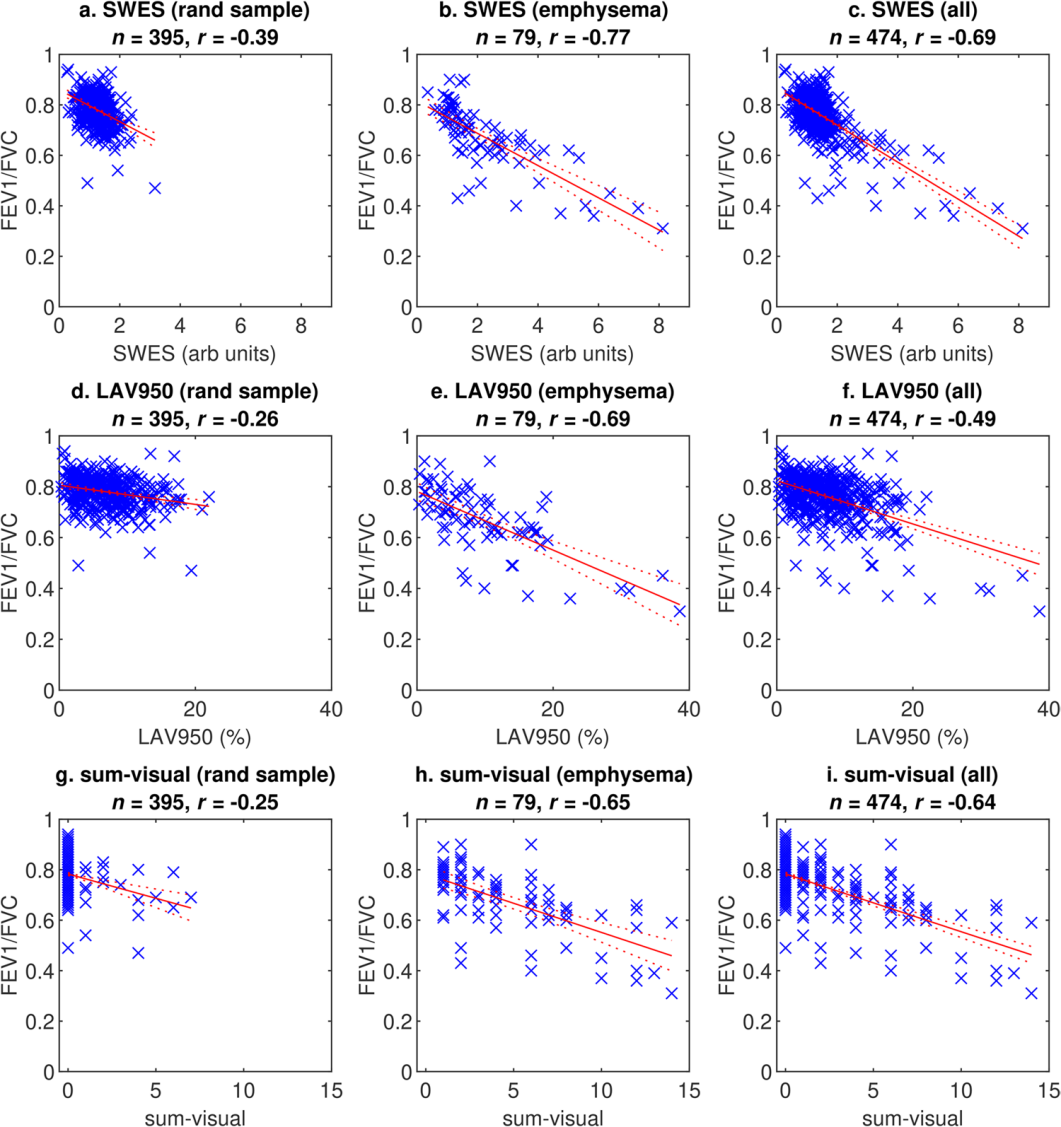
Scatter plots showing correlations with pulmonary function testing in random, emphysema, and total cohorts. (**a**–**c**) Slice-wise whole-lung emphysema score (SWES). (**d**–**f**) Low-attenuation volume below − 950 HU (LAV950). (**g**–**i**) Sum-visual. All correlations are statistically significant, *p* < 0.001

There was a strong inverse correlation with airway obstruction (FEV1/FVC) in the full cohort (*r* =  − 0.69, *p* < 0.001). The correlations between SWES, sum-visual, and LAV950, and FEV1/FVC, with pair-wise comparisons using Meng’s test are shown in Table 2. The correlation between SWES and FEV1/FVC was significantly stronger than the correlation between LAV950 and FEV1/FVC or between sum-visual and FEV1/FVC in all cohorts. Scatter plots illustrating the correlations are shown in Fig. 6.

**Table 2:** Pair-wise comparisons using Meng’s test regarding correlation with airway obstruction

	Random cohort (*n* = 395)	Emphysema cohort (*n* = 79)	Full cohort (*n* = 474)
*r* (SWES, FEV1/FVC); *r* (LAV950, FEV1/FVC) (*p*-value)	− 0.39; − 0.26 *p* = 0.001	− 0.77; − 0.69 *p* = 0.034	− 0.69; − 0.49 *p* < 0.001
*r* (SWES, FEV1/FVC); *r* (sum-visual, FEV1/FVC) (*p*-value)	− 0.39; − 0.25 *p* = 0.007	− 0.77; − 0.65 *p* = 0.002	− 0.69; − 0.64 *p* = 0.007
*r* (LAV950, FEV1/FVC); *r* (sum-visual, FEV1/FVC) (*p*-value)	− 0.26; − 0.25 *p* = 0.45	− 0.69; − 0.65 *p* = 0.26	− 0.49; − 0.64 *p* < 0.001

*r*, Pearson correlation coefficient. *p*-values using Meng’s test for differences in correlation coefficients in same sample. *SWES*, slice-wise whole-lung emphysema score; *LAV950*, percentage low-attenuation volume − 950 HU; *FEV1/FVC*, forced expiratory volume in 1 s divided by forced vital capacity

SWES was a better predictor of airway obstruction (defined as FEV1/FVC < 0.7) than either LAV950 (*p* = 0.007) or sum-visual (*p* = 0.004). The AUC for SWES, LAV950, and sum-visual for prediction of GOLD criteria for COPD (FEV1/FVC < 0.7) in the random cohort was 0.76 (95% CI 0.67–0.85), 0.61 (95% CI 0.50–0.72), and 0.62 (95% CI 0.54–0.70), respectively (see Fig. 5b).

##### Diffusing capacity

DLcoPred% was available in 61 and 328 participants in the emphysema and random cohort, respectively (see Table 3). In the emphysema cohort, SWES, sum-visual, and LAV950 were correlated to DLcoPred% (*r* =  − 0.74, *r* =  − 0.74, and *r* =  − 0.52, respectively, each *p* < 0.001). In the random cohort, with low emphysema frequency, SWES and sum-visual were weakly correlated to DLcoPrc (*r* =  − 0.21, *p* < 0.001 and *r* =  − 0.20, *p* < 0.001, respectively), while LAV950 was not correlated to DLcoPrc (*r* = 0.06, *p* = 0.27).

**Table 3:** Pair-wise comparisons using Meng’s test regarding correlation with diffusion capacity in percent of predicted

	Random cohort (*n* = 328)	Emphysema cohort (*n* = 61)	Full cohort (*n* = 389)
*r* (SWES, DLcoPred%); *r* (LAV950, DLcoPred%) (*p*-value)	− 0.21; 0.06 *p* < 0.001	− 0.74; − 0.52 *p* < 0.001	− 0.53; − 0.22 *p* < 0.001
*r* (SWES, DLcoPred%); *r* (sum-visual, DLcoPred%) (*p*-value)	− 0.21; − 0.20 *p* = 0.41	− 0.74; − 0.74 *p* = 0.49	− 0.53; − 0.54 *p* = 0.34
*r* (LAV950, DLcoPred%); *r* (sum-visual, DLcoPred%) (*p*-value)	0.06; − 0.20 *p* = 0.001	− 0.52; − 0.74 *p* = 0.002	− 0.22; − 0.54 *p* < 0.001

*r*, Pearson correlation coefficient. *p*-values using Meng’s test for differences in correlation coefficients in same sample. *SWES*, slice-wise whole-lung emphysema score; *LAV950*, percentage low-attenuation volume − 950 HU; *DLcoPred%*, carbon monoxide diffusion capacity in percent of predicted

The correlation with DLcoPred% was significantly stronger for SWES than for LAV950 in all validation cohorts (Meng’s test *p* < 0.001). Compared to sum-visual, the correlation was approximately equal in all cohorts (all *p* > 0.05).

## Discussion

In this study, a slice-wise whole-lung CT emphysema score was developed to obtain a method for the rapid identification of emphysema suitable for population-based large cohorts. We compared SWES with quantitative CT and the sum of regional visual emphysema scores. SWES was a significantly better approximation of the readers’ visual score than LAV950 and correlated significantly more strongly with pulmonary function testing than either sum-visual or LAV950.

Emphysema is visible in CT imaging as low-density regions and is an important predictor of mortality and morbidity in COPD, independent of lung function [2, 6–8]. The development of quantitative CT metrics for emphysema in recent decades parallels attempts to include the additional value of CT imaging in COPD models in especially research and clinical trials [4–8].

A threshold-based emphysema score such as LAV950 is a reasonable quantitative metric for emphysema, especially in cohorts with advanced disease [6–8, 10, 22]. However, in cross-sectional cohorts, there is considerable overlap in LAV950 between subjects with and without visual emphysema [17]. Furthermore, the readers’ visual emphysema estimation has been shown to provide additional predictive value, even in models that include thresholding [8].

The output measure of LAV950—the fraction of low-attenuation volume—is appealing as it may be interpreted as the proportion of lung parenchyma affected by emphysema. However, in diffusely distributed lung diseases, there is generally no clear cutoff in CT images between healthy and affected parenchyma [23], and the delineation based on attenuation alone is oversimplified. Low-density pixels can appear, for example, because of air-trapping, hyperinflated lungs, and image noise, phenomena that may be distinguished from emphysema by experienced readers.

The difficult delineation of emphysema also makes 3D CNN architectures, which are computationally logical for thin-slice CT data, challenging to apply with detailed visual scores as ground truth. 3D CNN may be applied on a global score basis such as PFT [22], but fine-grained global scores that truly represent visual emphysema are even more difficult to obtain than slice-wise scores. Instead, to address the continuous aspect of emphysema, we developed SWES as an aggregated score for each lung on a slice-wise basis. Acquisition of quality annotations is a challenge for supervised machine learning in radiology [24]. Multiple image comparisons by a large number of trained readers and approximate sorting enabled the creation of detailed training data.

We present two major results: First, SWES is a good approximation of the radiologist’s assessment, which shows that the algorithm measures what the radiologist identifies as emphysema. Second, SWES is a better predictor of chronic airway obstruction than either the reader’s visual score or LAV950. In addition, the correlation with reduced diffusing capacity was stronger compared to LAV950 and equal compared to visual scores. The absence of correlation between LAV950 and DLcoPred% in the random cohort is likely caused by the overlap in LAV950 between participants with and without emphysema and the low number of participants with severe emphysema in the random cohort.

The improved performance in predicting obstruction compatible with COPD may be explained by the greater granularity of SWES and the absence of the inter-observer variations inevitable in visual scoring [17, 25, 26]. While readers estimated the emphysema extent on a 4-point scale in three regions in each lung, SWES is an aggregated continuous score for each 0.6-mm slice of each lung, using 10-point scale training data. The improved performance than that of LAV950 indicates that counting low-attenuation pixels does not gather complete information regarding emphysema in CT images [8, 17].

Most previous studies using deep learning to detect emphysema in chest CT images have used smoking cohorts with a high frequency of COPD [22, 27–29]. Given the aim of the study, an important feature was the cross-sectional test cohort with low emphysema frequency. Comparison with a study by Singla et al illustrates the association between airway obstruction and visual emphysema scoring [22]. While Singla et al used PFT results as image labels and showed that the machine learning method also predicted visual emphysema, we took the opposite approach, using visual emphysema labels and showing that we could also predict the physiological airway obstruction [22].

### Limitations

The results indicate that the algorithm performs well with fixed imaging parameters in the validation cohort from the main SCAPIS study, although the reconstruction parameters were different in the training dataset. However, the differences in magnitude, as seen in Table 1, indicate that, similarly to LAV950, the magnitude of SWES is highly dependent on the reconstruction parameters and cannot be directly compared using different settings. For routine clinical use or with other image parameters, additional training to equalize the output would be necessary, but the demonstrated principle of an aggregated visual slice-wise score is valid.

Although we demonstrate that SWES correlates to visual emphysema scoring, the specific image features that the algorithm detects are unknown. Airways thickening, the other main component of COPD, was not assessed. The airway involvement as seen in CT also has clinical predictive value [30, 31] and should be included in future work.

The SWES scale, developed using ordinal data from radiologists’ annotations, is arbitrary and has no fixed reference, which makes score interpretation more difficult. The emphysema type, which may add predictive value, was not assessed in the study, but could be assessed with further developments.

In conclusion, SWES is a quantitative emphysema score for CT imaging that avoids the disadvantages of threshold-based scores and is correlated more strongly with reference tests than LAV950 and visual scores. Aggregated slice-wise emphysema quantification is especially suited for pulmonary research use in large-scale cross-sectional CT multi-center image cohorts.

### Supplementary Information

Below is the link to the electronic supplementary material.

Supplementary file1 (PDF 1.02 MB)

Supplementary file2 (ZIP 31 KB)

## Abbreviations

CNN: Convolutional neural network
COPD: Chronic obstructive pulmonary disease
DLcoPred%: Post-bronchodilator carbon monoxide diffusing capacity in percent of predicted
eCRF: Electronic case report form
FEV1/FVC: Forced expiratory volume in 1 s divided by forced vital capacity
LAV950: Percentage low-attenuation value below − 950 HU
PFT: Pulmonary Function Test (spirometry)
ROC: Receiver operating characteristics
SCAPIS: Swedish CArdiopulmonary bioImage Study
Sum-visual: Sum of regional visual emphysema grading in one participant
SWES: Slice-wise whole-lung emphysema score

## Declarations

### Guarantor

The scientific guarantor of this publication is Mats Lidén, MD, Assoc Prof.

### Statistics and biometry

One of the authors (ML) has significant statistical expertise.

### Study subjects or cohorts overlap

Concerning machine learning CT emphysema assessment, the validation cohort has not been previously reported. Related to the present work, the subjects in the development cohort have previously been reported by Vikgren et al [17] and Lidén et al [18].

### Methodology

• Prospective inclusion

• Cross-sectional

• Single inclusion site
