# Supplementary material for: Machine learning slice-wise whole-lung CT emphysema score correlates with airway obstruction
Source: Eur Radiol. 2023 Aug 8;34(1):39–49. doi: 10.1007/s00330-023-09985-3 (PMC10791709; doi:10.1007/s00330-023-09985-3)
Supplement: Supplementary file 1 — Supplementary file1 (PDF 1.02 MB) [file 330_2023_9985_MOESM1_ESM.pdf]

Supplemental figures

Supplemental Fig S1: Image label refinement. (a) Multi-reader multi-split annotations in 1 cm chunks. (b) After z-direction median filtering. (c) Sorting and refinement to ten-degree emphysema scale. (Modified from Lidén et al. Multi-Reader–Multi-Split Annotation of Emphysema in Computed Tomography. J Digit Imaging 33, 1185–1193 (2020). https://doi.org/10.1007/s10278-020-00378-2)

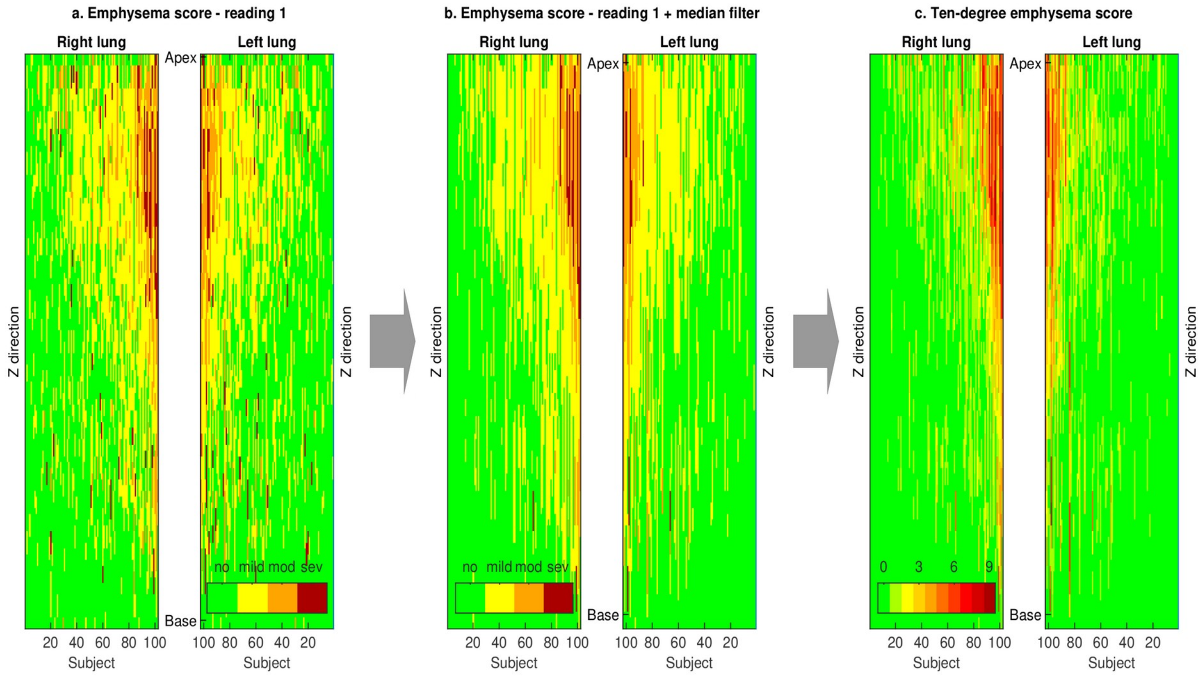

Supplemental Fig S2: Screenshot from annotation application.

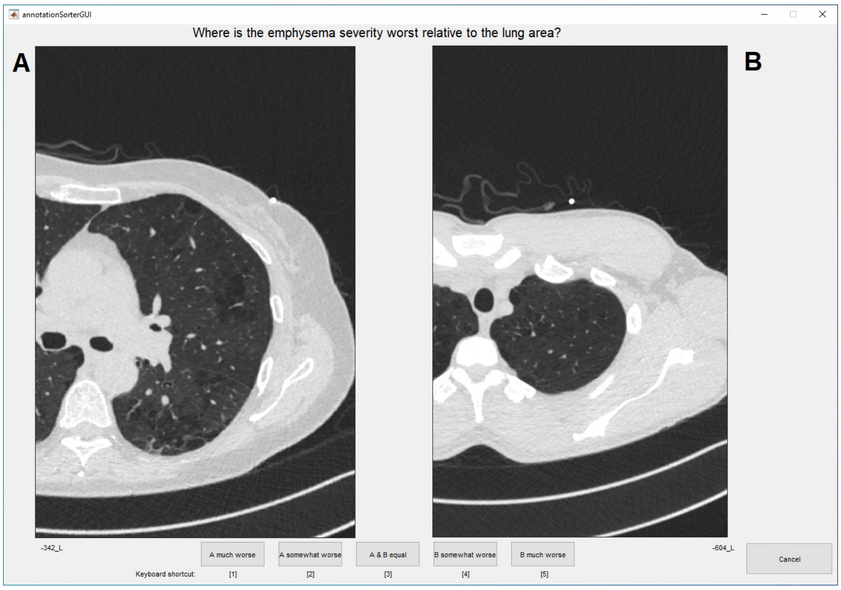

Supplemental Fig S3: Emphysema score expansion. The granularity of the 4-degree emphysema scale in the training dataset was increased to 10-degrees using within-class approximate sorting and reclassification.

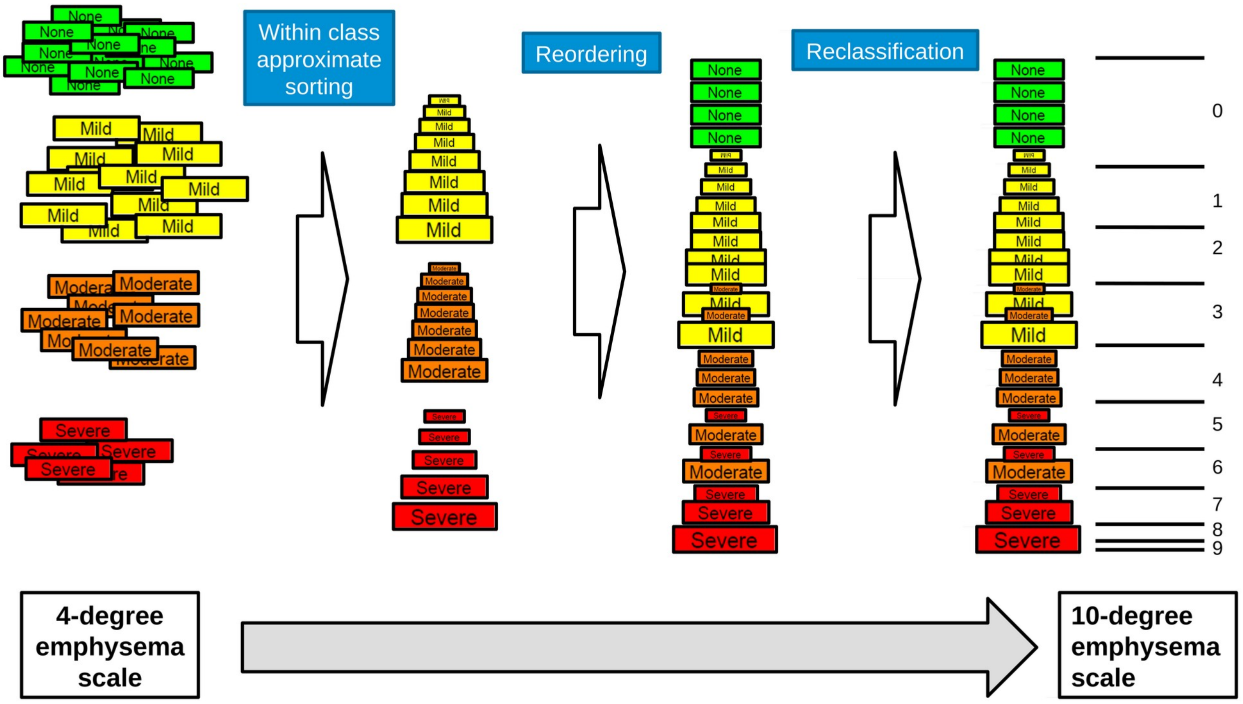

Supplemental Fig S4: CT image preprocessing in the training set, starting and ending with the segmented lung mask. For prediction, only segmentation, contrast windowing, cropping, and resizing were used.

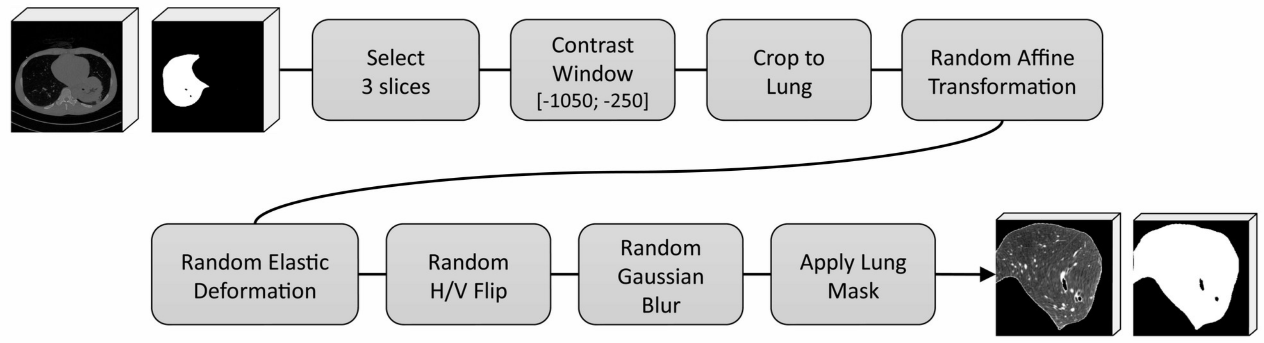

Supplemental Fig S5: Scatter plot demonstrating excellent correlation between LAV950 using Siemens AI Rad Companion and using SWES segmentations.

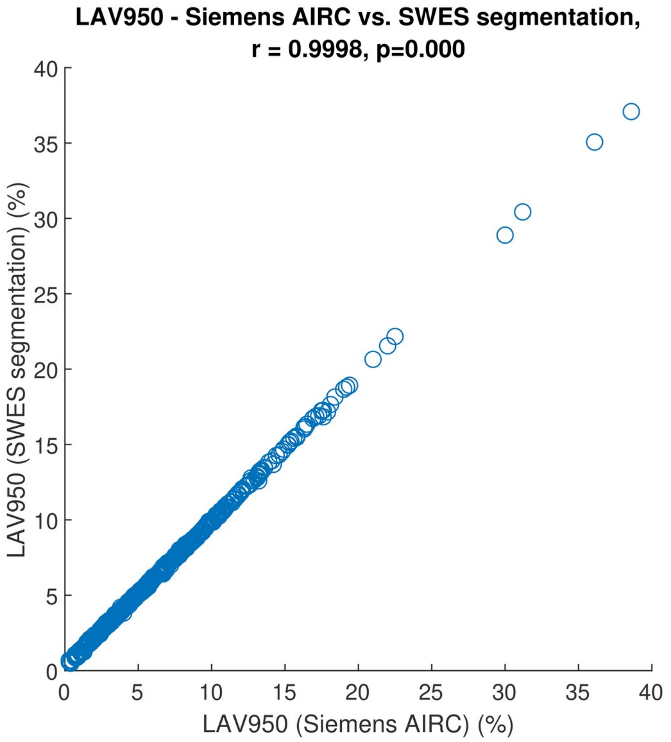

Supplement

Appendix A: Visual emphysema score expansion

Detailed slice-wise emphysema annotations were acquired in a three-step process, see supplemental Fig S1-S3. In the first step each centimeter of each lung was classified according to a four-degree emphysema scale. The median filtering in the second step removed outliers illustrated in supplemental Fig S1a, resulting in a smooth craniocaudal distribution of image labels, as expected for emphysema. In the third step the granularity of the emphysema labels was expanded from a four-degree scale to ten degrees.

To refine the four-degree emphysema scale into a ten-degree scale, approximate sorting using multiple comparisons was used in the third step. Sixteen readers were each shown 1,078 pairs of axial lung images. Each pair showed the right or left lung from different random z-locations and cases within the same label category after median filtering (mild, moderate or severe). For each pair, the reader assessed which image displayed the highest emphysema grade. The rationale for approximate sorting with multiple comparisons was that consistent scoring using a detailed scale is challenging, while the comparison of the relative severity between two displayed images was considered easier. A screenshot from the annotation application is shown in Supplemental Fig S2.

The 17,248 comparisons provided by the 16 readers were used in creating a refined ten-degree scale with emphysema annotations for each centimeter of each lung separately. The multiple comparisons provided by the readers were used for approximate sorting using a modified Borda counting algorithm, where “much worse” was weighted doubled compared to “somewhat worse”. Image slices that were mostly scored as worse emphysema grades were sorted as more severe, whereas slices with lower scores were downgraded, see Supplemental Figure S3.

In detail, the reordering of the annotations into a combined 10-degree scale using the approximately sorted lists in each category was performed in the following way: The top 7% of SEVERE emphysema slices received score 9, the following 24% score 8 and the following 38% score 7. Score 6 consisted of the following 24% of SEVERE slices and the top 7% of MODERATE slices, score 5 of the following 24% of MODERATE slices and the lowest 7% of SEVERE slices. Score 4 consisted of the mid 38% of MODERATE slices. Score 3 consisted of the following 24% of the MODERATE slices and the top 7% of the MILD slices and score 2 of the following 24% of the MILD slices. Score 1 consisted of the mid 38% of the MILD slices. Score 0 consisted of the lowest 31% of the MILD slices and all slices annotated as NONE.

Appendix B. CT-Scan Pre-processing

Lung Segmention

Lung segmentation and identification are necessary steps to extract the meaningful scan region and to focus only on the scan regions of interest. We therefore developed an unsupervised algorithm able to extract the right and left lungs separately. It worked reasonably well on almost all cases in

the training set, with manual corrections introduced where necessary. The algorithm was composed of two steps.

First a mask of both lungs is generated with a combination of thresholding and morphological cleaning. The scan is threholded with t =-200HU. The resulting mask is filtered with a 3D median kernel (3x3x3 voxels) followed by a morphological closing and opening with a ball shaped structuring elements with radius 3 voxels. Afterwards, as the threshold extract air-rich region, the masks at this stage contains essentially the air-background and the lungs, connected by the trachea. Therefore, the lungs can be extracted by identifying objects and keeping the second largest one as the largest is the background.

Secondly, the right and left lungs are identified by, first, sequentially eroding 5 times with a ball of radius 1 the lungs mask obtained in step 1 in order to create two objects. Then the objects are identified and the two largest are kept assuming they represents the lungs. Afterwards, each of the two objects are sequentially dilated three times with a ball of radius 3 to re-grow the object approximately to its original size. The lungs are then identified as being the right or the left one by observing where their mass center lies. Finally, because the sequential dilation tends to yield soft boundaries of the lung, the final right or left lung mask is obtained by taking the intersection between the sharp lungs mask of step 1 and the sequentially dilated one obtained in step 2. Note that to speed up the computation time, the lung identification is performed on a downsampled version of the mask obtained in step 1. The scan is compressed by a factor n· and the final mask is expanded by a factor 1/n·.

In the external validation using data from the main SCAPIS Gothenburg cohort, the slice-wise predictions were applied to all slices of the segmented lung. The slice-wise whole-lung emphysema score (SWES) was constructed as the average emphysema score in each lung and slice, weighted by the segmented lung area. In 10/474 cases, where the deterministic segmentation algorithm failed to identify the right and left lungs separately, corrected segmentations were applied.

Data augmentation

Upon data loading, online data augmentation is applied: first, to enrich the data and reduce overfitting on our limited dataset; second, to force the model to base its decision on the lung texture rather than shape, since the lesions are more present at the top and bottom of the lung. In our augmentation strategy, three slices are first randomly selected from the 17 slices of the chunk. Then a contrast window of [−1050; −250] Hounsfield units is applied to focus on the feature of interest. With the lung mask, the chunk is cropped to the lung and resized to 512 × 512 pixels. Afterwards, the chunk is augmented by applying one or more random affine transformations (rotation, scale, shear, and translation). Then, elastic deformation is applied to the chunk, which is then randomly flipped horizontally and vertically. Afterward, the chunk is randomly blurred with a Gaussian kernel. Finally, the chunk is multiplied with the mask to retain only the content of the lung. For validation/test chunks in the development cohort, the online transformation is restricted to selecting three slices (the 4th, 9th, and 14th), cropping to the lung, resizing, and applying the lung mask.

Data Imbalance Compensation

With only a few examples of serious emphysema lesions, there is a risk that the models' optimizations may not push the models to focus on those rare serious lesions. We thus need to encourage the network to focus more on those rare but serious emphysema lesions. Two methods are explored and used together: oversampling, in which the rare cases are presented more often during the optimization process; and weighted loss, in which the network is penalized more on the rare cases, see Supplemental Table S1.

Supplemental Table S1: Data imbalance compensation

	Label 0	Label 1	Label 2	Label 3	Label 4	Label 5	Label 6	Label 7	Label 8	Label 9
**Original subsample N chunks**	2404	468	228	120	77	40	21	21	9	4
**Oversampled N chunks**	2404	1404	912	600	462	280	168	189	108	100
**Penalization factor**	1	3	4	5	6	7	8	9	12	25

Note: Each chunk in the training set consisted of 17 CT slices with 0.6 mm slice thickness covering 1 cm in the z-direction.

Appendix C: Validation of automatic LAV950 metrics

The LAV950 metrics used in the study ($LAV950_{AIRC}$) were obtained in a fully automatic workflow using commercially available software, AI-Rad Companion Chest CT (AIRC, Siemens Healthineers). To check for possible segmentation problems in the fully automated $LAV950_{AIRC}$ workflow, the results were compared to LAV950 measurements using the segmentation algorithm from the pre-processing step of the SWES algorithm including corrections ($LAV950_{SWES}$).

The LAV950 output was almost identical using the two approaches, which indicates that the segmentations without manual verification in the fully automatic workflow were valid. The Pearson correlation coefficient between $LAV950_{AIRC}$ and $LAV950_{SWES}$ was 0.9998, see supplemental Fig S5. The Bland-Altman limits of agreement for $LAV950_{AIRC}$ and $LAV950_{SWES}$ were 0.0±0.2 percentage points.
